# Integration of the B-Cell Receptor Antigen Neurabin-I/SAMD14 Into an Antibody Format as New Therapeutic Approach for the Treatment of Primary CNS Lymphoma

**DOI:** 10.3389/fonc.2020.580364

**Published:** 2020-11-12

**Authors:** Moritz Bewarder, Maximilian Kiefer, Clara Moelle, Lisa Goerens, Stephan Stilgenbauer, Konstantinos Christofyllakis, Dominic Kaddu-Mulindwa, Natalie Fadle, Evi Regitz, Frank Neumann, Markus Hoth, Klaus-Dieter Preuss, Michael Pfreundschuh, Lorenz Thurner

**Affiliations:** ^1^ José Carreras Center for Immuno- and Gene Therapy, Saarland University Medical Center, Homburg, Germany; ^2^ Internal Medicine I, Saarland University Medical Center, Homburg, Germany; ^3^ Biophysics, CIPMM, Saarland University, Homburg, Germany

**Keywords:** B-cell receptor antigens, primary central nervous system lymphoma, neurabin-I/SAMD14, BAR-body, auto-antigens

## Abstract

Recently, neurabin-I and SAMD14 have been described as the autoantigenic target of approximately 66% of B-cell receptors (BCRs) of primary central nervous system lymphomas (PCNSL). Neurabin-I and SAMD14 share a highly homologous SAM domain that becomes immunogenic after atypical hyper-N-glycosylation (SAMD14 at ASN339 and neurabin-I at ASN1277). This post-translational modification of neurabin-I and SAMD14 seems to lead to a chronic immune reaction with B-cell receptor activation contributing to lymphoma genesis of PCNSLs. The selective tropism of PCNSL to the CNS corresponds well to the neurabin-I and SAMD14 protein expression pattern. When conjugated to Pseudomonas Exotoxin A (ETA´), the PCNSL reactive epitope exerts cytotoxic effects on lymphoma cells expressing a SAMD14/neurabin-I reactive BCR. Thus, the reactive epitopes of SAMD14/neurabin-I might be useful to establish additional therapeutic strategies against PCNSL. To test this possibility, we integrated the PCNSL-reactive epitope of SAMD14/neurabin-I into a heavy-chain-only Fab antibody format in substitution of the variable region. Specific binding of the prokaryotically produced SAMD14/neurabin-I Fab-antibody to lymphoma cells and their internalization were determined by flow cytometry. Since no established EBV-negative PCNSL cell line exists, we used the ABC-DLBCL cell lines OCI-Ly3 and U2932, which were transfected to express a SAMD14/neurabin-I reactive BCR. The SAMD14/neurabin-I Fab antibody bound specifically to DLBCL cells expressing a BCR with reactivity to SAMD14/neurabin-I and not to unmanipulated DLBCL cell lines. Eukaryotically produced full-length IgG antibodies are well established as immunotherapy format. Therefore, the PCNSL-reactive epitope of SAMD14/neurabin-I was cloned into a full-length IgG1 format replacing the variable domains of the light and heavy chains. The IgG1-format SAMD14/neurabin-I construct was found to specifically bind to target lymphoma cells expressing a SAMD14/neurabin-I reactive B cell receptor. In addition, it induced dose-dependent relative cytotoxicity against these lymphoma cells when incubated with PBMCs. Control DLBCL cells are not affected at any tested concentration. When integrated into the Fab-format and IgG1-format, the PCNSL-reactive epitope of SAMD14/neurabin-I functions as **B**-cell receptor **A**ntigen for **R**everse targeting (BAR). In particular, the IgG1-format BAR-body approach represents a very attractive therapeutic format for the treatment of PCNSLs, considering its specificity against SAMD14/neurabin-I reactive BCRs and the well-known pharmacodynamic properties of IgG antibodies.

## Introduction

Primary diffuse large B-cell lymphoma (DLBCL) of the central nervous system (CNS), also called primary central nervous system lymphoma (PCNSL) (1), is a rare disease with an annual incidence rate of 0.47 cases per 100.000 population (2), accounting for <1% of all non-Hodgkin lymphomas (3). Genetic profiling studies suggest that the normal counterpart of PCNSL cells are late germinal center (GC) exit B-cells with a gene expression profile characteristic of both GC B-cells and activated B-cells (ABC) (1, 4). In accordance with this, PCNSLs show rearranged immunoglobulin genes, ongoing somatic hypermutation, and persistent B cell lymphoma 6 (BCL6) activity but lack terminal B-cell differentiation resulting in a fixed IgM/IgD phenotype (5–8). Frequent mutations in the B-cell receptor (BCR) and Toll-like receptor 9 (TLR9) pathways (9, 10) with transcriptional upregulation of NF-kB, overrepresentation of the autoimmunity-linked immunoglobulin gene VH4-34 (5, 11) and persistent expression of functional BCRs despite ongoing somatic hypermutation (7), all indicate an important role of antigenic BCR stimulation in the pathogenesis of PCNSL (12). This seems particularly true for primary vitreoretinal lymphomas which display an even more restricted immunoglobulin gene repertoire (11). PCNSLs are confined to the CNS, limited exclusively to the brain, spinal cord, leptomeninges, and eyes. This selective tropism may be caused by proteins that are expressed exclusively in the CNS stimulating only B-cells residing in the CNS (12, 13). Importantly, the CNS counts as immune sanctuary along with eyes and testes, allowing for lymphoma development under reduced immunosurveillance (14, 15).

First-line treatment of PCNSL is divided into induction therapy followed by a consolidation regimen (16). High initial response rates of up to 86% can be achieved with induction therapies incorporating the agents methotrexate, rituximab, thiotepa, and cytarabine (MATRix) (17). With the results of the randomized phase 3 trial “HOVON 105/ALLG NHL 24,” which found no clear benefit of the addition of rituximab to chemotherapy, the role of rituximab in first-line treatment of PCNSLs is controversial (18). High-dose chemotherapy with autologous stem cell transplantation (ASCT) is thought to be an effective, safe, and feasible consolidation strategy (19, 20). Whole brain radiotherapy (WBRT) is another effective treatment option but associated with long-term side effects like impairment of higher cognitive functions (20, 21). Even though initial response rates are high, almost half of responders are bound to relapse and about one-third of the patients are primary refractory (22–24). Additionally, conventional immunochemotherapy is accompanied by many side effects like cytopenia and immunoglobulin deficiency, putting patients at high risk of infections and bleeding events. New and specifically targeted strategies are therefore urgently needed as treatment options for PNCSL.

We recently identified the sterile α-motif domain containing protein 14 (SAMD14) and the neural tissue-specific F-actin binding protein I (neurabin-I) as antigenic targets of the BCR from 66% (8/12 recombinant BCRs, cloned from primary tissue biopsies) of PCNSLs (12). SAMD14 and neurabin-I are primarily expressed in the CNS and share a highly homologous domain (SAM) in which the BCR binding epitope was identified. This SAM-domain was found to be post-translationally modified (N-hyperglycosylated) only in PCNSL patients with BCRs of SAMD14 and neurabin-I reactivity. It is speculated that this posttranslational modification results in a chronic immune reaction leading to lymphoma development over time (12). When coupled to the pseudomonas exotoxin A (ETA), the PCNSL-binding epitope of SAMD14/neurabin-I (hereafter referred to only as neurabin-I) confers cytotoxic effects against DLBCL cells transfected with neurabin-I-specific BCRs *in vitro*. This therapeutic approach has been termed “BAR” for “**B**-cell receptor **A**ntigen for **R**everse targeting” (12, 25).

The aim of this work is to integrate the PCNSL-specific BAR neurabin-I into an antibody format to generate a neurabin-I BAR-body and to test its functionality. To achieve this, neurabin-I was first integrated into an antibody fragment (antigen binding fragment = Fab) and later into a full length IgG1 antibody for assessment of binding properties and therapeutic potential.

## Materials and Methods

### Bacteria, Cell Lines, and Cell Culture

The DLBCL cell line U2932 was kindly provided by the Dr. Senckenberg Institute of Pathology of the Goethe University Hospital Frankfurt. HEK 293T and OCI-Ly3 cells were purchased from DSMZ (Braunschweig, Germany). For authentication, concordance of the VH gene sequences with published sequences was demonstrated by PCR analysis and sequencing. Cells were cultured in RPMI 1640 medium (Pan Biotech, Aidenbach, Germany), supplemented with 4 mmol glutamine and 10% FCS. Peripheral blood mononuclear cells (PBMCs) were prepared by Ficoll density centrifugation (1,500 rpm for 30 min) and used on day 2 after preparation without stimulation. DH5α competent *Escherichia coli* (*E. coli*) were obtained from Thermo Scientific™ (Waltham, MA, USA) and used for general cloning and sub-cloning. TG1 *E. coli* were used for expression of the heavy-chain-only Fab-format neurabin-I BAR-body.

### Expression of a Neurabin-I Reactive BCR in OCI-Ly3 and U2932 Cells

To our knowledge there are no EBV-negative PCNSL cell lines which could be tested for neurabin-I autoreactivity and used for further studies. As substitute, two DLBCL cell lines, OCI-Ly3 and U2932, were transfected with a BCR with neurabin-I specificity cloned from a DNA sample of a PCNSL patient. For cloning we used a modified pRTS-1 expression vector which comprised following components: a heavy chain variable region (VH), heavy chain constant regions CH1-CH4, TM1, and TM2 as transmembrane regions, a cytoplasmic tail, a furin + 2A sequence, a light chain variable region (VL), and the light chain constant region (12, 26). VH and VL genes were derived from the neurabin-I reactive BCR of a PCNSL patient and obtained by digesting cryosections with 2 μl of proteinase K (Roche PCR grade) for 4 h at 55°C, followed by heat-inactivation at 95°C for 10 min. The subsequent semi nested PCRs were performed as described previously (12, 27, 28). PCR products of the PCNSL-derived VH and VL were re-extended according to corresponding immunoglobulin germline genes.

For transfection, 2 × 10^7^/ml OCI-Ly3 and U2932 cells were washed three times with FCS-free RPMI-1640. A Gen Pulser (Biorad) with a 0.2 cm cuvette was used to transfect 2 × 10^6^ cells with 5 μg plasmid DNA (140 V, 30 ms pulses). Cells were then put on ice and cultured in RPMI-1640 medium with 20% FCS. Selection of stably transfected cell lines was done with hygromycin at 250 μg/ml. Variable region gene PCRs were used to confirm successful transfection. Western blot and flow cytometry with anti-Flag antibodies were used to show expression of the Flag-tagged BCR.

### Cloning Strategy for the Heavy-Chain-Only Fab-Format Neurabin-I BAR-Body

A modified pCES1 vector was used for the assembly of recombinant heavy-chain-only Fab-format neurabin-I BAR-bodies (29), comprising neurabin-I in substitution for the heavy chain variable domain (VH) and a CH1 domain. The light chain region was removed from the pCES1 vector by digesting 2 µg vector with 10 U of the restriction enzymes HindIII and AscI (Thermo Scientific™, Waltham, MA, USA) for 1 h at 37°C. For DNA blunting, the vector was incubated with DNA Polymerase I, Large (Klenow) fragment (Thermo Scientific™, Waltham, MA, USA) for 1 h at 37°C and blunt ends were ligated with 5 U T4-DNA ligase (Thermo Scientific™, Waltham, MA, USA) at 37°C over-night. The construct was coupled to a modified exotoxin A of *Pseudomonas aeruginosa* (ETA’) by digesting the processed pCES1 vector with the restriction enzymes NotI and EcoRI following ligation of the accordingly prepared ETA’ DNA fragment as described previously (30).

Three versions of the heavy-chain-only Fab-format neurabin-I BAR-body were cloned differing in the neurabin-I sequence selected to replace the variable region: version A (aa 1204–1324 of neurabin-I), version B (aa 1168–1285 of neurabin-I), version C (aa 1131–1250 of neurabin-I). Primers used are listed in **Table 1**. All versions contained the PCNSL binding neurabin-I epitope of 26 amino acids (aa 1226–1251) (12) at different positions from 5’ to 3’.

**Table 1:** Primers used for heavy-chain-only Fab-format neurabin-I BAR-bodies.

Heavy-chain-only Fab-format BAR-Body	Name	Sequence (5′-3′)
A	NRBI AA1204-NcoI-sense	CCA TGG CCA ATT TTA CCT TCA ATG AT
	NRBI AA1324-BstEII-antisense	GGT GAC CAT TTC CTT GAG TTT CTT
B	NRBI AA1168-NcoI-sense	CCA TGG CCA ACA CAT GGA TTA CAA AA
	NRBI AA1285-BstEII-antisense	GGT GAC CCC GAA TTC AGA TAC ATA CTG
C	NRBI AA1131-NcoI-sense	CCA TGG CCA ATG ACA GCC GGA AAG GA
	NRBI AA1251-BstEII-antisense	GGT GAC CCC AGA CTG TCC ATC ATC AAG

### Expression, Purification, and Detection of the Heavy-Chain-Only Fab-Format Neurabin-I BAR-Body

TG1 *E. coli* bacteria were transformed with the new vector comprising the heavy-chain-only Fab-format neurabin-I BAR-body coupled to ETA’. Recombinant soluble heavy-chain-only Fab BAR-bodies were expressed and purified as described previously (29, 31). In short, 50 µl of IPTG was added to 50 ml of TG1 bacteria (TY medium, cell density of 0.6 to 0.8 measured at 600 nm) for 4 h at 30°C and then centrifuged at 4,000 rpm for 15 min. After lysing, His-tagged and ETA’-coupled heavy-chain-only Fab-format neurabin-I BAR-bodies were purified by immobilized metal affinity chromatography (IMAC) using Talon beads (Takara Bio USA, Inc., Mountain View, CA, USA). Proteins were eluted with 150 mM imidazole for 5 min at room temperature and detected by western blot analysis using Mouse Anti-His Tag Recombinant Antibody (Qiagen, Hilden, Germany) and Goat Anti-Mouse IgG (H + L)-HRP Conjugate (Bio-Rad, Munich, Germany).

### Cloning Strategy for the IgG1-Format Neurabin-I BAR-Body

To assemble the sequence the IgG1-format neurabin-I BAR-body a pSfi FLAG-Tag expression vector was used. The pSfi FLAG-Tag vector is derived from the pEGFP-C1 vector of Clontech (Mountain View, CA, USA) from which the eGFP ORF was removed and replaced by a FLAG-Tag. A pSfi-cloned IgG1 sequence consisting of a VH, the heavy chain constant regions CH1-CH3, a Furin + 2A sequence, a VL, and the light chain constant region (26) served as template. VH and VL were exchanged with a sequence of similar length (approximately 120 amino acids) of neurabin-I (aa 1168–1285) containing the PCNSL reactive epitope (aa 1226–1251). Primers used are listed in **Table 2**. Restriction enzymes MunI and BstEII (Thermo Fisher Scientific, Waltham, USA) were used for insertion of the neurabin-I fragment into the VH region whereas AgeI and SmaI were used for insertion of the neurabin-I fragment into the VL region.

**Table 2:** Primers used for the IgG1-format neurabin-I BAR-body.

IgG1-format BAR-Body	Name	Sequence (5′-3′)
Heavy chain	NRBI AA1168-MunI-sense	CAA TTG AAC ACA TGG ATT ACA AAA
	NRBI AA1285-BstEII-antisense	GGT GAC CCC GAA TTC AGA TAC ATA CTG
Light chain	NRBI AA1168-AgeI-sense	ACC GGT AAC ACA TGG ATT ACA AAA
	NRBI AA-1285-SmaI-antisense	CCC GGG GAA TTC AGA TAC ATA CTG

### Expression, Purification, and Detection of the IgG1-Format Neurabin-I BAR-Body

The completely assembled IgG1-format neurabin-I BAR-body was transfected into HEK 293T cells for production. HEK 293T cells were cultivated to a confluence of about 50–70%. One microgram plasmid DNA was diluted in 100 µl RPMI1640, mixed with 3 µl of X-tremeGENE™ HP DNA Transfection Reagent (Sigma-Aldrich Chemie GmbH, Munich, Germany) and incubated at room temperature for 10 min. Afterwards the preparation was added to HEK 293T cell cultures in a drop by drop fashion.

Purification of the IgG1-format neurabin-I BAR-body from the supernatant was performed *via* anti-FLAG antibody affinity chromatography using the ANTI-FLAG^®^ M2 Affinity Gel (Sigma-Aldrich, St. Louis, USA). To purify 50 ml supernatant, 200 µl anti-FLAG M2-Affinity Gel was washed once with 1 ml PBS and then incubated with supernatant overnight at 4°C. The mixture was then centrifuged for 10 min at 2,500 rpm which was repeated a second time after discarding the supernatant in a new vessel for 3 min at 1,800 rpm. Three washing steps followed, each with 1 ml PBS and centrifugation was performed at 10,000 rpm for 30 s. Flag-tagged IgG1 neurabin-I BAR-bodies were eluted from the gel by adding 250 µl glycine pH 3 for 2 min with subsequent centrifugation at 10,000 rpm for 30 s. Supernatant containing the isolated IgG1 BAR-bodies was supplemented with 25 µl Na2HPO4. To exchange the IgG1 BAR-bodies into a different buffer system overnight dialysis against PBS was performed.

The BAR-body was detected by western blot analysis. Concentrated IgG1-format neurabin-I BAR-bodies were loaded on to a 10% SDS-PAGE and transferred to PVDF membrane using a transblot semi-dry transfer cell (Bio Rad). After blocking overnight at 4°C in PBS supplemented with 10% nonfat dry milk, transferred proteins were incubated with murine Monoclonal ANTI-FLAG^®^ M2 antibody (Sigma-Aldrich, St. Louis, USA) at 1:2,500 for 45 min at room temperature. The membrane was rinsed with TBS 5 times for 2 min each before and after it was incubated with Goat Anti-Mouse IgG (H+L)-HRP conjugate (Bio-Rad, Feldkirchen) at 1:3,000 for 45 min. The chemiluminescence reagent LumiGLO^®^ Reagent (Cell Signaling Technology, Frankfurt, Germany) was used for immunoblot detection.

### Flow Cytometric Binding Assays

All flow cytometric analyses were performed using a BD FACS Canto Flow Cytometer and data was analyzed with WinMDI Software version 2.3 (Purdue University Cytometry Laboratories). Binding of heavy-chain-only Fab-format neurabin-I BAR-bodies to neurabin-I reactive BCRs was tested as follows: 5 × 10^6^ OCI-Ly3 cells transfected with a neurabin-I reactive BCR were incubated with 5 μg/ml of each BAR body (versions A, B, and C) for 30 min at 4°C, washed with PBS and stained with 5 µl of Penta-His-Allophycocyanin-Antibody (Qiagen, Hilden, Germany) for 30 min at 4°C. OCI-Ly3 cells not transfected with the recombinant neurabin-I reactive BCR served as control cells and MAZ, the BCR target of the leukemic cells from a CLL patient, as control antigen (12).

Internalization assays were performed for version B of the Fab-format neurabin-I BAR-bodies (32). 5 × 10^6^ OCI-Ly3 cells transfected with a neurabin-I reactive BCR were incubated with 5 μg/ml of the Fab-format neurabin-I BAR body (version B) for 30 min at 4°C. After a washing step in PBS, cells were incubated at 37°C for 30 min, fixed with 500 μl of 2% paraformaldehyde (PFA) for 15 min at 4°C, and permeabilized with 800 μl of 0.5% saponin. Intracellular staining of internalized Fab-format BAR-body was performed using 5 µl of Penta-His-APC-Antibody (Qiagen, Hilden, Germany) for 30 min at 4°C.

To determine binding properties of IgG1-format neurabin-I BAR-bodies to target cells, 5 × 10^6^ U2932 cells transfected with a neurabin-I reactive BCR were incubated with 10 µg/ml IgG1-format neurabin-I BAR-bodies for 30 min, washed in PBS, and stained with murine Monoclonal ANTI-FLAG^®^ M2 antibody for 30 min at 4°C (Sigma-Aldrich, St. Louis, USA). After another washing step in PBS, final staining was performed with goat anti-mouse IgG (H+L), APC for 30 min at 4°C (Thermo Scientific™, Waltham, MA, USA). IgG1 format BAR-bodies incorporating LRPAP1, an irrelevant BCR antigen of mantle cell lymphomas, served as controls.

### Cytotoxicity Assays

Cytotoxicity of the constructs was determined by LDH release assay (“Cytotoxicity Detection Kit” by Roche). 5 × 10^3^ OCI-Ly3 and U2932 lymphoma cells with or without neurabin-I reactive BCR were placed in each well.

Version B of ETA’-coupled Fab-format neurabin-I BAR-body and IgG1-format neurabin-I BAR-body were applied at concentrations of 10, 5, 2.5, and 1.25 µg/ml. As Controls the constructs MAZ-ETA’ and an IgG1-format BAR-body incorporating the B-cell receptor antigen LRPAP1 (25) or no construct were used.

To facilitate antibody-dependent cell-mediated cytotoxicity (ADCC), PBMCs were added to target cells and IgG1-format BAR-bodies at an effector/target ratio of 10:1, corresponding to 5 × 10^4^ PBMCs per well.

Specific lysis was determined as (experimental lysis – spontaneous lysis)/(maximum lysis − spontaneous lysis) × 100. Maximum lysis was determined after adding 10% Triton X-100. Lactate dehydrogenase (LDH) was measured according to the protocol of the LDH assay kit (Roche, Mannheim, Germany). ELISA read-out was done using a Victor II microplate reader (PerkinElmer, Rodgau, Germany).

All experiments were performed in triplicate. Values (OD at 490 nm or specific lysis in %) are given as statistical mean of three experiments ± standard error of the mean.

## Results

### Cloning and Expression of the Heavy-Chain-Only Fab-Format Neurabin-I BAR-Bodies

**Figure 1:**
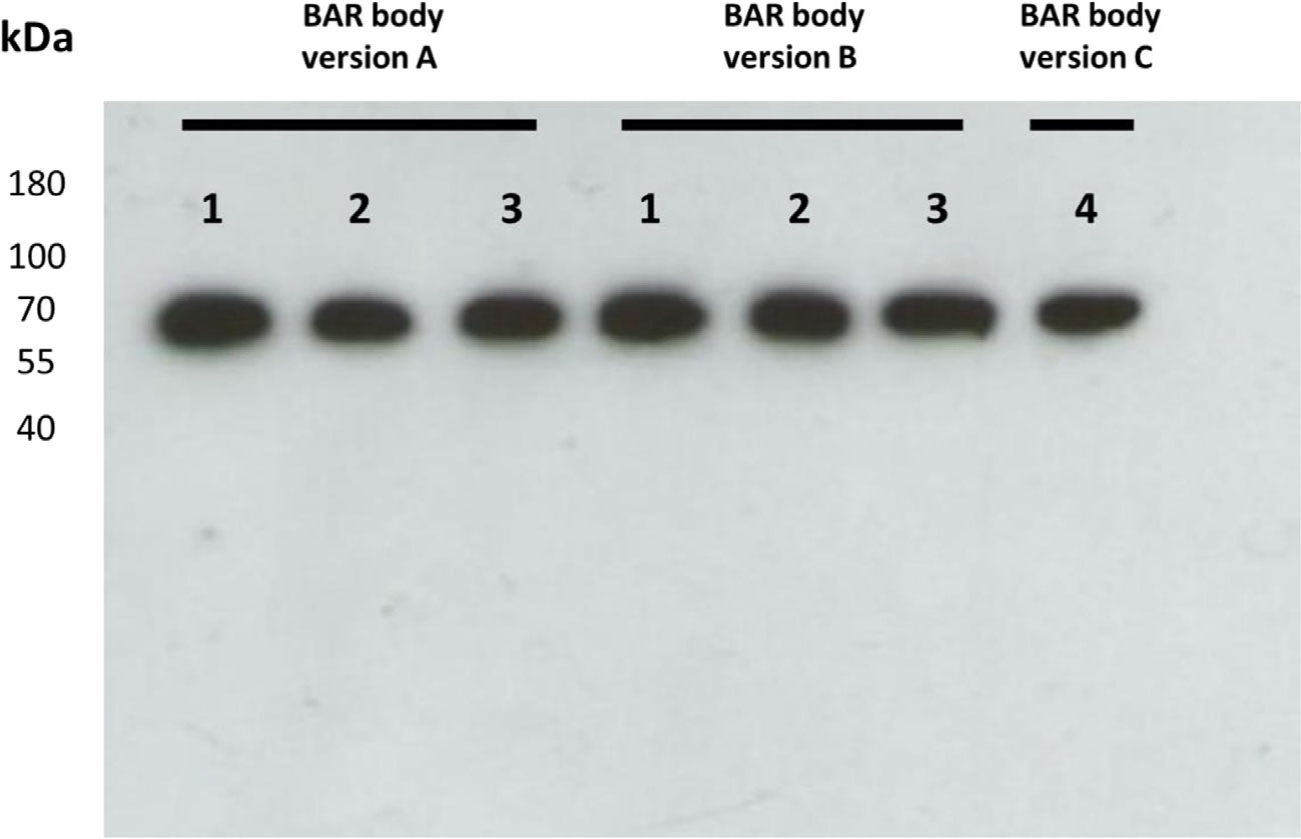
Western blot of heavy-chain-only Fab-format neurabin-I BAR bodies after expression in TG1 E. coli bacteria. Fab-format neurabin-I BAR-bodies versions A, B [three clones each (1–3)] and C (single clone 4) were produced. All Fab-format BAR bodies had the expected molecular weight of approximately 67 kDa corresponding to 628 amino acids (1,884 base pairs).

We aimed to clone and express three versions of a BAR-body construct that integrates the BCR-binding epitope of neurabin-I into a heavy-chain-only Fab-format. The variable region of a Fab-antibody is replaced by the BCR-binding epitope of neurabin-I (aa 1226–1251) to create the BAR region of the Fab-format BAR-body (**Supplementary Figures 1** and **2**). In order to mimic the molecular mass and conformation of a normal Fab-antibody, the epitope region was complemented by amino acids either at the NH2 (version A), the COOH (version C), or both ends (version B). The heavy-chain-only Fab-format neurabin-I BAR bodies in their versions A, B, and C were coupled to ETA’ to mediate cytotoxicity to target cells (**Supplementary Figure 1**). The amino acid sequence enumeration refers to isoform 3 of neurabin-I (uniprot accession number: Q9ULJ8-3). Western blot analysis shows successful expression of heavy-chain-only Fab-format neurabin-I BAR bodies after expression in TG1 *E. coli* bacteria. Versions A and B were produced as three clones each (1–3) and BAR-Body C was produced as single clone (clone 4). All Fab-format BAR bodies had the expected molecular weight of approximately 67 kDa corresponding to 628 amino acids (**Figure 1**).

### Identification of a Fab-Format BAR-Body Version With Highly Selective Binding Properties to Lymphoma Cells Expressing Neurabin-I Reactive BCRs

**Figure 2:**
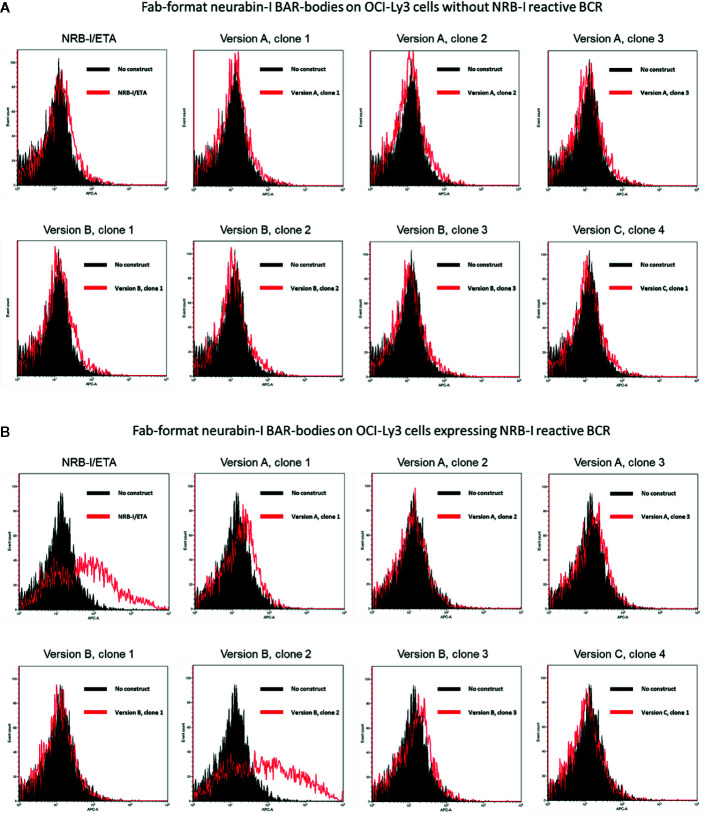
Identification of a Fab-format BAR-body incorporating neurabin-I with binding capacity to DLBCL cells expressing a neurabin-I reactive BCR. Fab-format BAR bodies were generated in three different versions (version A, version B, and version C; see **Supplementary Figure 1**). **(A)** All three versions were tested for binding to OCI-ly3 cells that were not transfected with a patient-derived neurabin-I reactive BCR. No unspecific binding was detected. **(B)** Clone 2 of version B showed binding to OCI-ly3 cells transfected to express a neurabin-I reactive BCR similar to positive controls consisting of the immunotoxin NRB-I/ETA that has previously been shown to specifically bind to neurabin-I reactive BCRs.

All three Fab-format BAR-body versions were tested for binding to OCI-ly3 cells transfected to express a patient-derived neurabin-I reactive BCR. As a control, all BAR-bodies were first tested against unmanipulated OCI-Ly3 cells; no binding could be observed (**Figure 2A**). All BAR- bodies were then tested against OCI-ly3 cells expressing a neurabin-I reactive BCR. As a positive control, the immunotoxin NRB-I/ETA was measured since it was previously shown to specifically bind to neurabin-I reactive BCRs (12). NRB-I/ETA bound indeed to OCI-ly3 cells expressing a neurabin-I reactive BCR (**Figure 2**). Of the BAR body clones, clone 2 of version B bound to OCI-ly3 cells expressing a neurabin-I reactive BCR, whereas all other clones did not bind (**Figure 2B**).

We tested the binding of clone 2 version B in a second independent set of experiments against an irrelevant antigen (MAZ) coupled to the pseudomonas exotoxin A (MAZ/ETA). In the control experiments, no binding could be observed against unmanipulated OCI-Ly3 cells (**Supplementary Figure 3A**) but again we found specific binding of clone 2 version B but not the irrelevant antigen MAZ against OCI-ly3 cells expressing a neurabin-I reactive BCR (**Supplementary Figure 3B**).

Finally, surface (**Supplementary Figure 4A**) and intracellular (**Supplementary Figure 4B**) staining of OCI-ly3 cells transfected to express neurabin-I reactive BCRs with heavy-chain-only Fab-format neurabin-I BAR-bodies shows internalization of Fab-format BAR-bodies after 30 min incubation at 37°C. MAZ/ETA was used as control (left histograms of **Supplementary Figures 4A, B**).

We conclude that clone 2 version B binds specifically to OCI-ly3 cells expressing a neurabin-I reactive BCR. Therefore, the Fab-format BAR-body of clone 2, version B was selected for further evaluation and a new batch of constructs was produced (**Supplementary Figure 5**).

### Heavy-Chain-Only Fab-Format Neurabin-I BAR-Body Mediates Killing of Lymphoma Cells Expressing a Neurabin-I Reactive BCR

**Figure 3:**
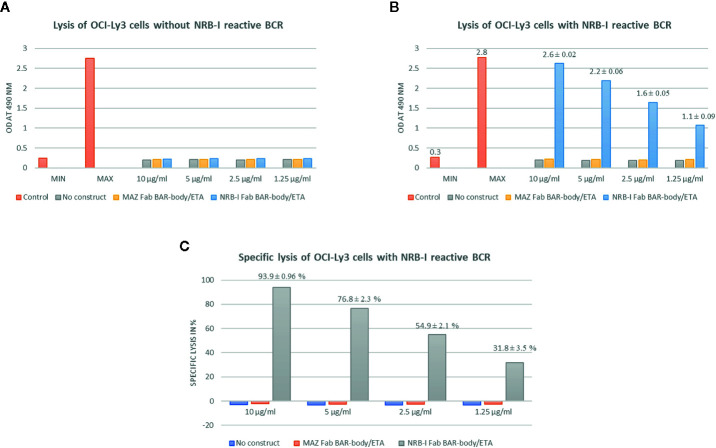
Heavy-chain-only Fab-format neurabin-I BAR-body induced specific cytotoxicity as measured by LDH release. MAZ/ETA, an irrelevant antigen (BCR target of the leukemic cells of a CLL patient) linked to pseudomonas exotoxin A, was used as control. All experiments were performed in triplicate. **(A)** Heavy-chain-only Fab-format neurabin-I BAR-body incubation with OCI-ly3 DLBCL cells at different concentrations (1.25–10 µg/ml) results in no LDH release indicating no unspecific Fab-format BAR-body induced cytotoxicity. **(B)** Heavy-chain-only Fab-format neurabin-I BAR-body at different concentrations (1.25–10 µg/ml) confers cytotoxicity to OCI-ly3 DLBCL cells transfected with neurabin-I reactive BCRs. Maximum LDH release is reached at approximately 10 µg/ml. **(C)** Fab-format neurabin-I BAR-bodies mediate specific lysis (calculated as described in the material and methods section) in a dose dependent manner, starting with approximately 30% at 1.25 µg/ml and reaching >90% at 10 µg/ml.

Heavy-chain-only Fab-format neurabin-I BAR-body-induced specific cytotoxicity was measured by LDH release. No construct, MAZ/ETA and unmanipulated OCI-Ly3 cells were used as controls (**Figure 3**). Incubation of heavy-chain-only Fab-format neurabin-I BAR-bodies (of clone 2 version B) with un-transfected OCI-ly3 DLBCL cells at different concentrations (1.25–10 µg/ml) resulted in no LDH release indicating no unspecific Fab-format BAR-body-induced cytotoxicity (**Figure 3A**). In contrast, heavy-chain-only Fab-format neurabin-I BAR-bodies of clone 2 version B conferred cytotoxicity to OCI-ly3 DLBCL cells transfected with neurabin-I reactive BCRs. Fab-format BAR-body concentrations from 1.25 to 10 µg/ml were tested. Maximum LDH release was reached at approximately 10 µg/ml (**Figure 3B**). Fab-format BAR-body-induced lysis in relation to minimal and maximal lysis (specific lysis) is dose dependent, starting with approximately 30% at 1.25 µg/ml and reaching >90% at 10 µg/ml (**Figure 3C**) compared to maximal lysis by triton.

### Generation of an IgG1-Format Neurabin-I BAR-Body

**Figure 4:**
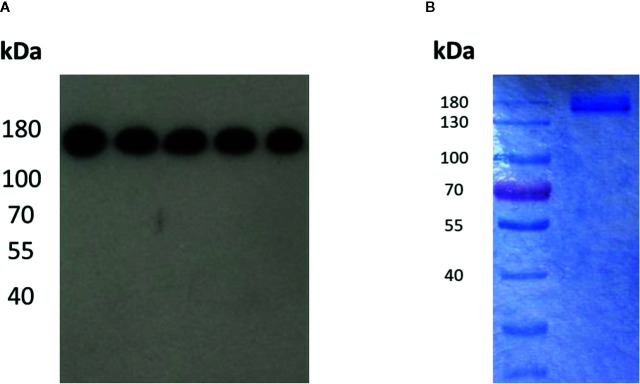
Detection of the IgG1-format neurabin-I BAR-body in Western Blot analysis using anti-Flag antibodies **(A)** and in Coomassie Blue staining **(B).** Both analyses show the estimated molecular weight of the IgG1-format BAR-body of approximately 150 kD comparable to normal IgG antibodies.

After determining the appropriate conformation of the BAR-region in Fab-format antibodies, we applied these findings to the generation of an IgG1-format BAR-body. We exchanged the variable regions of an IgG1 antibody for neurabin-I and adjacent amino acids as performed in the BAR-region of the Fab-format BAR-body, version B (**Supplementary Figure 6**). IgG1-format BAR-bodies were detected by Western Blot analysis using anti-Flag antibodies (**Figure 4A**) and by Coomassie Blue staining (**Figure 4B**). Both analyses confirmed the estimated molecular weight of the IgG1-format BAR-body of approximately 150 kD which is comparable to the molecular weight of IgG antibodies.

### Highly Selective Binding of IgG1-Format Neurabin-I BAR-Bodies to Lymphoma Cells Expressing Neurabin-I Reactive BCRs

**Figure 5:**
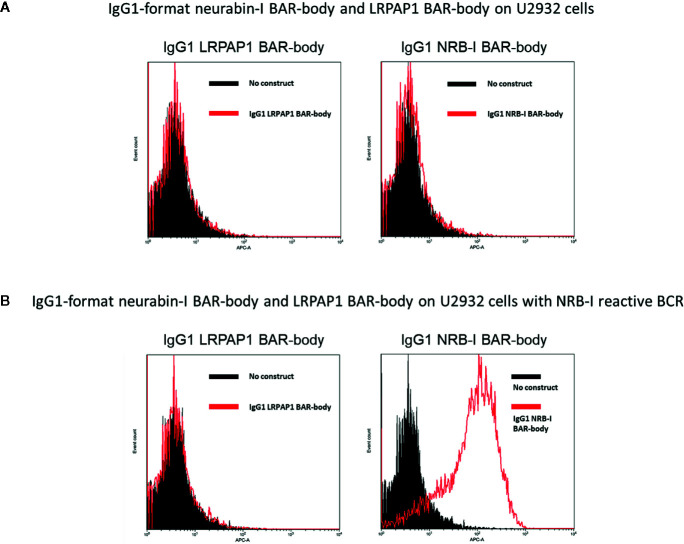
U2932 cells **(A)** and U2932 cells transfected with a neurabin-I reactive BCR **(B)** were stained with 10 µg/ml IgG1-format neurabin-I BAR-body. Mouse anti-Flag antibody followed by APC-conjugated anti-mouse antibody were used for detection. IgG1-format BAR-bodies incorporating LRPAP1, an irrelevant BCR antigen of mantle cell lymphomas, served as controls (left histograms of **A, B**).

To test whether IgG1-format neurabin-I BAR-bodies bind to lymphoma cells expressing neurabin-I reactive BCRs, U2932 cells (**Figure 5A**) and U2932 cells transfected with a neurabin-I reactive BCR (**Figure 5B**) were stained with 10 µg/ml IgG1-format neurabin-I BAR-body. Selective binding of the IgG1-format neurabin-I BAR-bodies to lymphoma cells with neurabin-I reactive BCRs was observed (compare right-side histograms of **Figures 5A, B**). IgG1-format BAR-bodies incorporating LRPAP1, an irrelevant BCR antigen of mantle cell lymphomas (25), served as control (left histograms of **Figures 5A, B**).

### IgG1-Format Neurabin-I BAR-Bodies Confer Selective, ADCC-Mediated, Cytotoxic Effects to Lymphoma Cells

We tested if IgG1-format neurabin-I BAR-bodies induce PBMC-mediated specific cytotoxicity against lymphoma cells expressing a BCR with neurabin-I reactivity. No construct, IgG1-format BAR-bodies incorporating LRPAP1 (25) or U2932 cells expressing native BCRs were used as controls.

**Figure 6:**
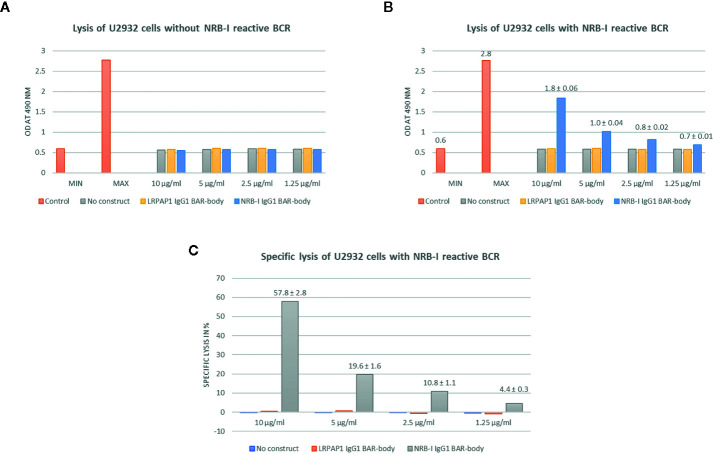
IgG1-format neurabin-I BAR-body induced PBMC-mediated specific cytotoxicity as measured by LDH release. IgG1-format BAR-bodies incorporating LRPAP1, an irrelevant BCR antigen of mantle cell lymphomas, were used as controls. All experiments were performed in triplicate. **(A)** IgG1-format neurabin-I BAR-body at different concentrations (1.25–10 µg/ml) incubated with U2932 DLBCL cells and PBMCs (E:T ratio of 10:1) results in no LDH release indicating no unspecific IgG1-format BAR-body induced cytotoxicity. **(B)** IgG1-format neurabin-I BAR-body (concentrations 1.25–10 µg/ml) confers dose-dependent and PBMC-mediated cytotoxicity to U2932 DLBCL cells transfected with neurabin-I reactive BCRs at an effector to target ratio of 10:1.**(C)** IgG1-format neurabin-I BAR-bodies confer specific lysis to U2932 cells transfected to express a neurabin-I reactive BCR (PBMC-mediated ADCC). LDH release is mediated in a dose-dependent manner, starting from 4% at 1.25 µg/ml going up to 58% at 10 µg/ml.

IgG1-format neurabin-I BAR-bodies were tested at different concentrations (1.25–10 µg/ml). After incubation with unmanipulated U2932 DLBCL cells and PBMCs (E:T ratio of 10:1) no LDH release was observed indicating no unspecific IgG1-format BAR-body-induced cytotoxicity (**Figure 6A**). When applying IgG1-format neurabin-I BAR-body to U2932 DLBCL cells transfected with neurabin-I reactive BCRs and PBMCs (E:T ratio of 10:1), dose-dependent PBMC-mediated cytotoxicity was observed (**Figure 6B**).

Specific lysis is mediated in a dose-dependent manner, starting from 4% at 1.25 µg/ml and going up to 58% at 10 µg/ml (**Figure 6C**) compared to the triton control.

## Discussion

We developed a molecule resembling an IgG1 antibody, that contains the BCR-binding epitope of the common PCNSL antigens SAMD14 and neurabin-I instead of variable regions to target BCRs with specificity for neurabin-I. This molecule, termed IgG1-format neurabin-I BAR-body, showed binding capacity only to lymphoma cells expressing a BCR with neurabin-I reactivity and exerted cytotoxic effects exclusively on lymphoma cells expressing a BCR of this specificity. As shown recently, more than 50% of all PCNSLs express a BCR with reactivity against neurabin-I (12) rendering more than half of all PCNSLs theoretically susceptible to neurabin-I BAR-body therapy.

The BCR of B-cell lymphoma cells is considered to be an ideal target for therapeutic approaches since it has a unique variable region (also called idiotype) distinguishing it from BCRs of normal B-cells and it is expressed abundantly on lymphoma cells. Different strategies have been developed to exploit the distinctiveness of the BCR of B-cell lymphomas. Anti-idiotype antibodies were the first therapeutics to be developed and tested with moderate success. They were either collected from animals immunized with the BCR of a patient´s lymphoma (33–36) or produced by patient vaccination with processed B-cell receptors of lymphoma cells (37, 38). More recently, Ronald Levy et al. developed a treatment approach termed peptibody, where small peptides are selected using a phage display library to develop anti-idiotype peptides that are affixed to an IgG Fc protein (39). The major drawback of anti-idiotype therapies developed so far is that they have to be produced individually on a patient by patient basis. Therapies incorporating a common B-cell receptor antigen that is shared as the antigenic target of a large percentage of a given B-cell lymphoma entity, like the neurabin-I BAR-body in the treatment of CNS lymphomas, would overcome such problems.

The findings of Thurner et al. (12) differ from those published by Montesinos-Rongen, who describes the BCRs of PCNSLs as mostly polyreactive with some BCRs recognizing galectin-3 and other antigens expressed in the CNS (13, 40). Further studies investigating the reactivity of more recombinant PCNSL-derived BCRs using an identical methodological approach will have to resolve these conflicting results. Nevertheless, it is noteworthy that our treatment approach to integrate BCR antigens into antibody formats would be applicable to all antigens identified.

The described neurabin-I BAR-body mimics the molecular structure of an IgG1 antibody with the variable regions exchanged for the shared PCNSL BCR epitope of SAMD14 and neurabin-I. The PCNSL binding epitope of neurabin-I comprises 26 amino acids (aa 1226–1251) which is much shorter than immunoglobulin variable regions, normally consisting of 110–130 aa (12, 41). To match the size of immunoglobulin variable regions in the neurabin-I BAR-body, we elongated the PCNSL-binding epitope to 120 amino acids with adjacent neurabin-I amino acids creating the BAR region. At the start it was unknown how the addition of amino acids to the BCR binding epitope would influence its binding properties. Therefore, we used a heavy-chain-only Fab-format to test different BAR region formations, containing the PCNSL-binding epitope either at its beginning (aa 1204–1324 of neurabin-I), its middle (aa 1168–1285 of neurabin-I), or its end (aa 1131–1250 of neurabin-I). The amino acid sequence numeration refers to isoform 3 of neurabin-I. These three neurabin-I BAR regions, carrying the common epitope responsible for binding to the BCR of PCNSL at different positions, were produced prokaryotically as heavy-chain-only Fab-format BAR-bodies. These initial exploratory experiments were performed due to lack of information on how the integration of the neurabin-I epitope into an antibody format would influence its binding properties to neurabin-I specific BCRs. When the 26 aa comprising neurabin-I epitope is flanked by non-epitope regions to form a 120 aa neurabin-I BAR region (aa 1168–1285 of neurabin-I) its binding properties to PCNSL BCRs are preserved. The causes for the lack of binding capacity of the other two Fab-format BAR-body variations to PCNSL BCRs with neurabin-I reactivity are not clear. Accordingly, the eukaryotically produced, full-length IgG1 neurabin-I BAR-body incorporating the appropriate BAR region was also able to bind to DLBCL cells, that were genetically engineered to express a patient-derived BCR with neurabin-I reactivity. *In-vitro* LDH-release assays showed the ability of the Fab-format and IgG1-format neurabin-I BAR-bodies to selectively kill DLBCL cells expressing corresponding BCRs.

The glycosylation status of produced BAR-bodies has not been assessed. Mass spectrometry analysis might be able to determine the glycosylation status of integrated neurabin-I epitopes. But while it would be interesting to determine hyper N-glycosylation of the described BAR-bodies, we think it has no bearing on our experiments. In previous work it could be shown that PCNSL-derived BCRs reacted with both the normally glycosylated and the hyper N-glycosylated SAMD14/neurabin-I isoforms (12). The isoform-specific immune reaction may be mediated by CD4+ T cells, which stimulate non-isoform-specific neurabin-I reactive B cells.

It remains to be clarified whether the IgG format is suitable for the further development of BAR-integrating therapeutics. The concentrations that are needed for the reported neurabin-I BAR-body to reach approximately 50% specific lysis are higher than the concentrations that have been reported for rituximab (42). This difference may be attributed to different factors. First, the PCNSL-derived BCR was transfected to be expressed by DLBCL cell lines which also express their natural BCR possibly resulting in a reduced expression of the PCNSL BCR on cells. Secondly, the BAR-body construct targets the variable region of the BCR with its presumed cognate antigen. This BCR-antigen interaction has not been quantified yet, for example by determining the affinity of neurabin-I to the BCR by plasmon resonance imaging. A low affinity may be sufficient to contribute to lymphoma development but may also necessitate high concentrations of BAR-bodies incorporating the cognate BCR antigen.

Regarding the size of full-length IgG antibodies, it is thought to result in an impaired ability to pass the blood-brain barrier and therefore leading to reduced CNS antibody concentrations. Rubenstein et al reported rituximab concentrations of as low as 0.1% of serum antibody concentrations (43). The role of the IgG antibody rituximab in the first-line treatment of PCNSL has been controversial, but evidence from the IELSG trials indicate clinical efficacy (17) and there have been reports of complete remissions after i.v. rituximab administration in the relapse setting (44). Also, intrathecal application of BAR-bodies is a conceivable route of administration (45). We therefore think that the IgG format is appropriate for the treatment of PCNSLs but as long as there is no *in-vivo* data, this notion remains speculative.

Taken together, our study justifies further development of BAR-bodies as promising tools for the treatment of CNS lymphomas. Nevertheless, major challenges might arise that range from the identification of more PCNSL antigens over the penetration of the blood-brain barrier to escape mechanisms of malignant B-cells. When the B-cell receptor is targeted, these problems could result from genetic alterations, common to malignant B cells like somatic hypermutation, to reduce the affinity to a B-cell receptor antigen and therapeutic BAR approaches. Thus, *in-vivo* studies are needed to further investigate the efficacy and potential drawbacks of the IgG1-format neurabin-I BAR-body in the treatment of CNS lymphoma.

## Data Availability Statement

The original contributions presented in the study are included in the article/**Supplementary Material**, further inquiries can be directed to the corresponding author.

## Author Contributions

MB and LT wrote the manuscript. MB, LT, FN, K-DP, and MP designed and supervised the experiments. LG, CM, MK, ER, and NF performed the experiments. MB and LT are responsible for data analysis. SS, KC, DK-M and MH revised the manuscript. All authors contributed to the article and approved the submitted version.

## Funding

This work was supported by a grant from Wilhelm-Sander-Stiftung (a charity foundation in Munich, Germany).

## Conflict of Interest

The authors declare that the research was conducted in the absence of any commercial or financial relationships that could be construed as a potential conflict of interest.
