# Supplementary material for: Integration of the B-Cell Receptor Antigen Neurabin-I/SAMD14 Into an Antibody Format as New Therapeutic Approach for the Treatment of Primary CNS Lymphoma
Source: Front Oncol. 2020 Nov 12;10:580364. doi: 10.3389/fonc.2020.580364 (PMC7689012; doi:10.3389/fonc.2020.580364)
Supplement: Supplementary file 1 [file Table_1.docx]

***Supplemental material***


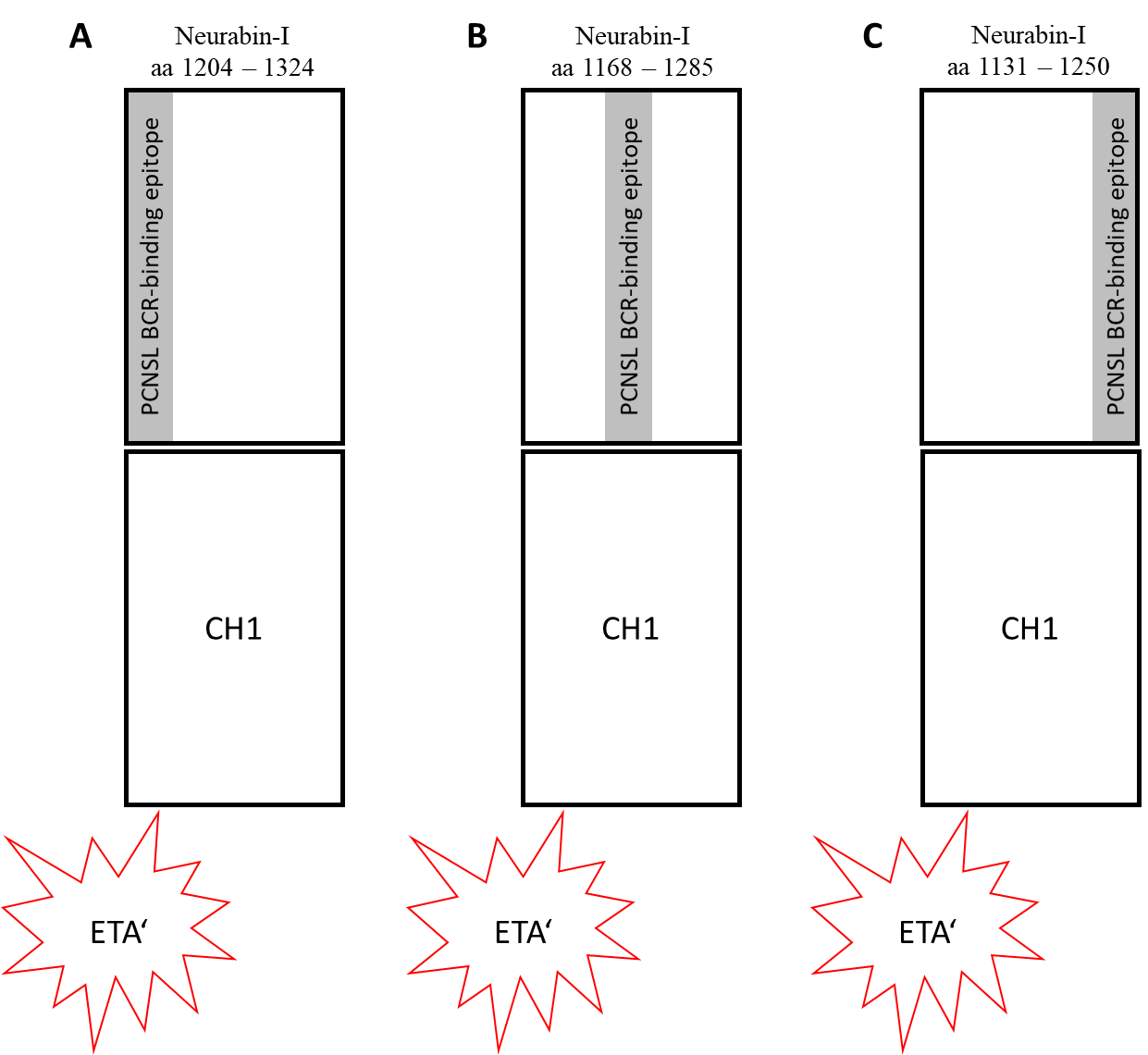


***Supplemental figure 1:*** Schematic of the heavy-chain-only Fab-format neurabin-I BAR bodies in their versions A, B and C coupled to ETA'. The grey bar represents the PCNSL-binding epitope of neurabin-I (aa 1226 – 1251). The amino acid sequence enumeration refers to isoform 3 of neurabin-I (https://www.uniprot.org). The constructs consist of the BAR region (neurabin-I epitope + adjacent regions) coupled to CH1 and Pseudomonas aeruginosa exotoxin A (ETA') as effector component. The length of the BAR region was chosen to mimic the immunoglobulin variable region. To this end, the 26 amino acids of the PCNSL-binding epitope of neurabin-I were extended either at the 5’ (A), the 3’ (C) or both ends (B).


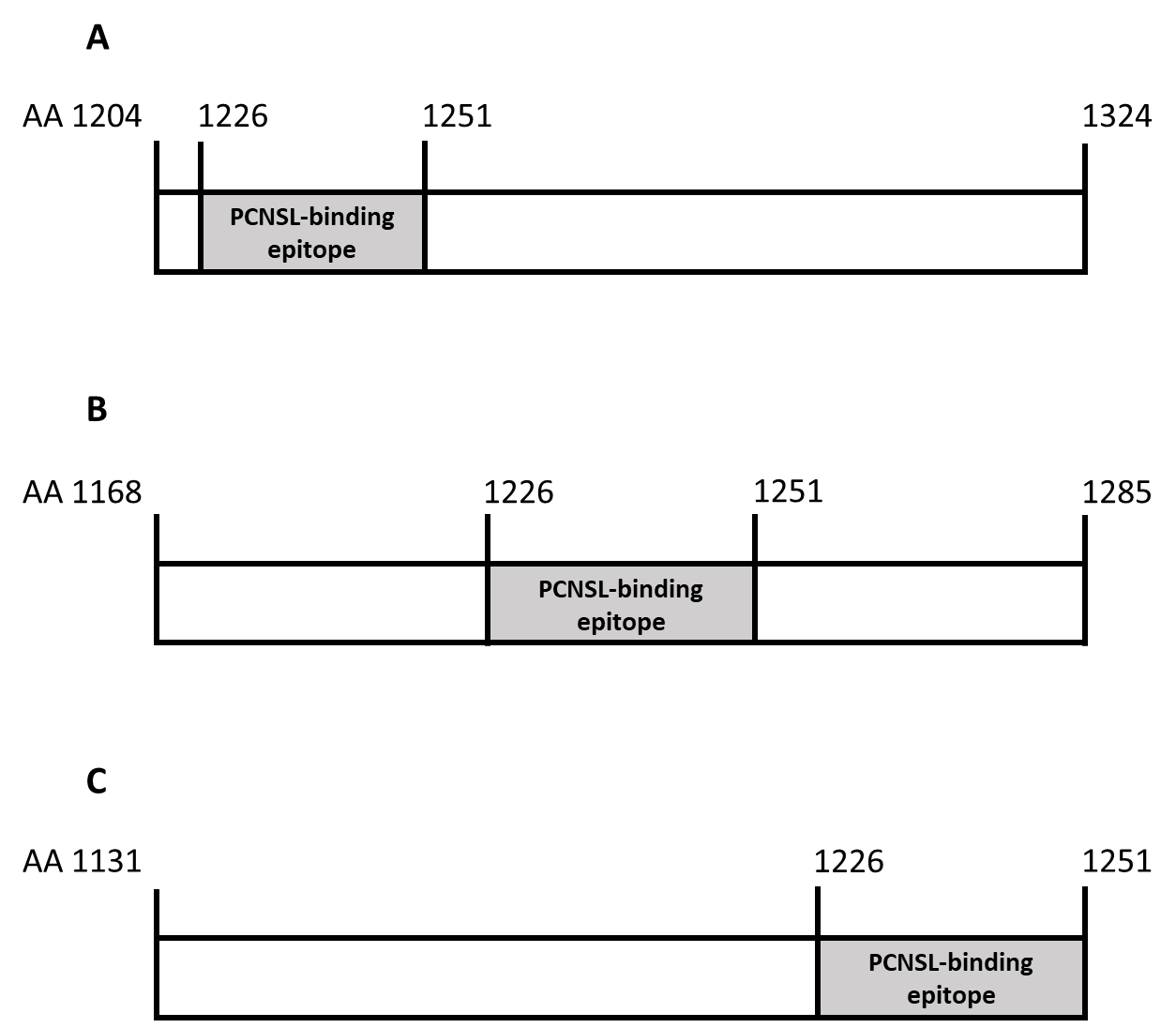


***Supplemental figure 2:*** Amino acid sequences of the BAR regions of the heavy-chain-only Fab-format neurabin-I BAR bodies in their versions A, B and C. The length of the BAR region was chosen to mimic the immunoglobulin variable region. The 26 amino acids of the PCNSL-binding epitope of neurabin-I (aa 1226 – 1251) were elongated either at the 5’ (A), the 3’ (C) or both ends (B) with adjacent amino acids of neurabin-I. The amino acid sequence enumeration refers to isoform 3 of neurabin-I (uniprot accession number: Q9ULJ8-3).


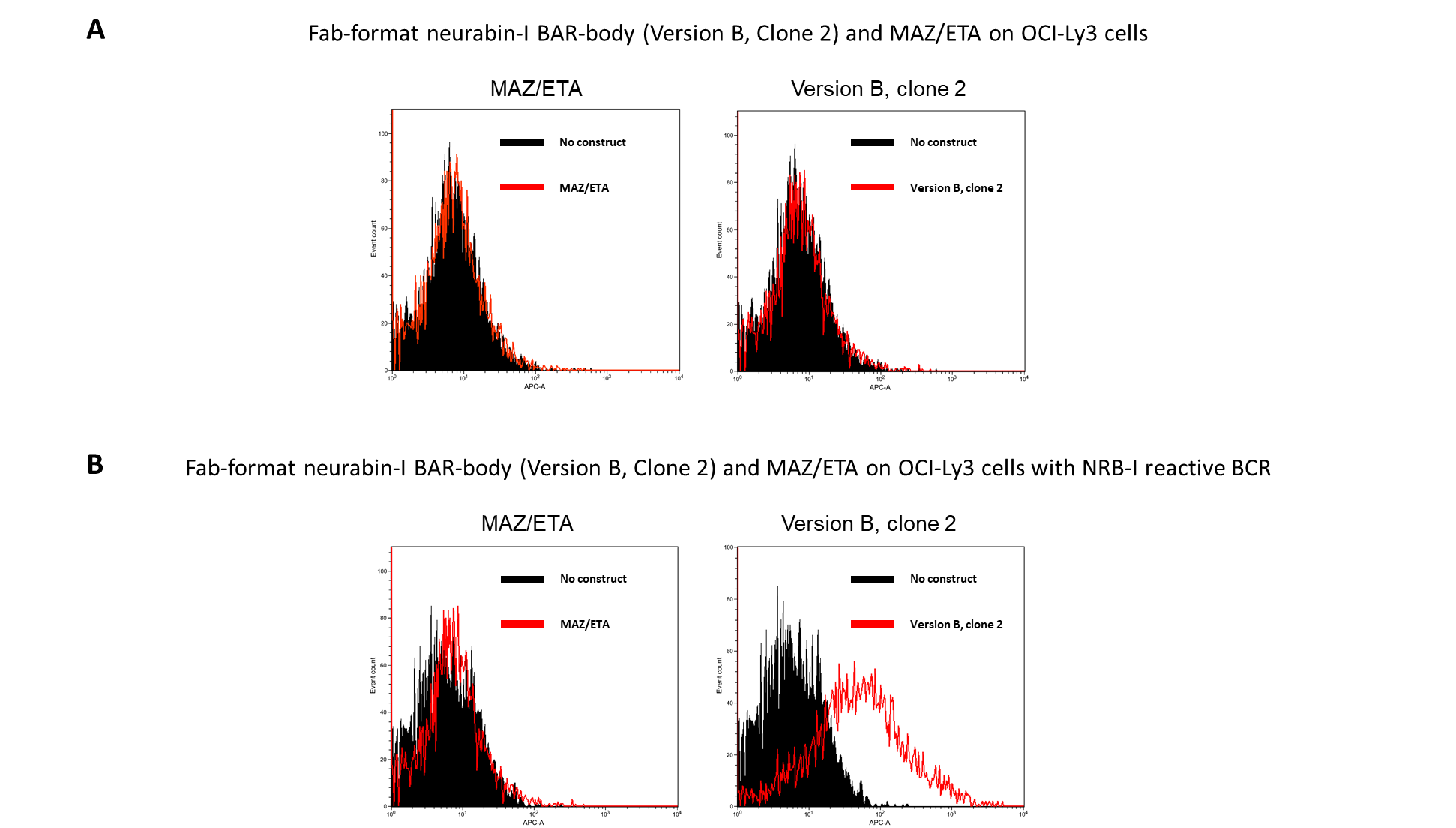


Supplemental figure 3: Heavy-chain-only Fab-format neurabin-I BAR-body (version B, clone 2) showed no binding to OCI-ly3 cells (A) as compared to OCI-ly3 cells transfected to express neurabin-I reactive BCRs (B). An irrelevant antigen (MAZ) coupled to the pseudomonas exotoxin A was used as control (left histograms of A and B).


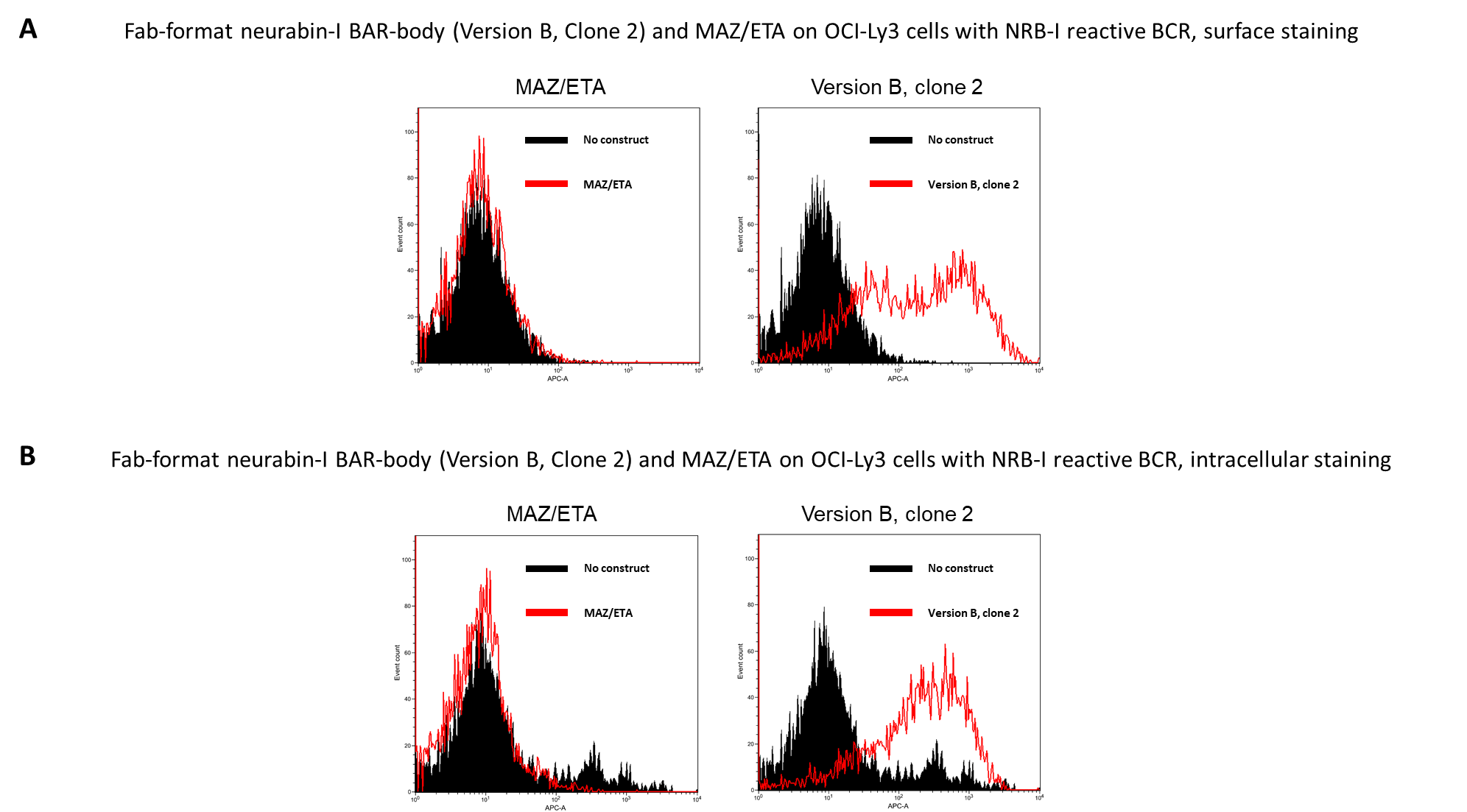


Supplemental figure 4: Surface (A) and intracellular (B) staining of OCI-ly3 cells transfected to express neurabin-I reactive BCRs with heavy-chain-only Fab-format neurabin-I BAR-bodies. Surface staining was performed after incubation for 30 minutes at 4°C. Intracellular staining was performed after incubation for 30 minutes at 4°C followed by 30 minutes incubation at 37°C before fixation and permeabilization. An irrelevant antigen (MAZ) coupled to the pseudomonas exotoxin A was used as control (left histograms of A and B).


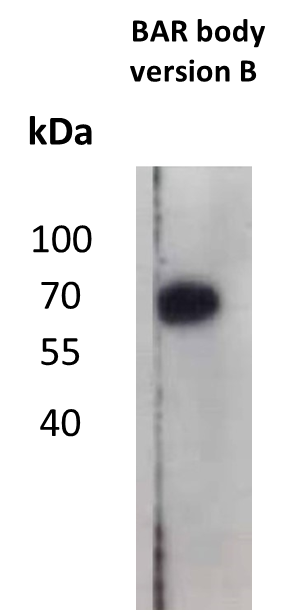


Supplemental figure 5: Western blot of heavy-chain-only Fab-format neurabin-I BAR body (version B, clone 2) after determination of binding capacity to OCI-ly3 cells expressing neurabin-I reactive BCRs.


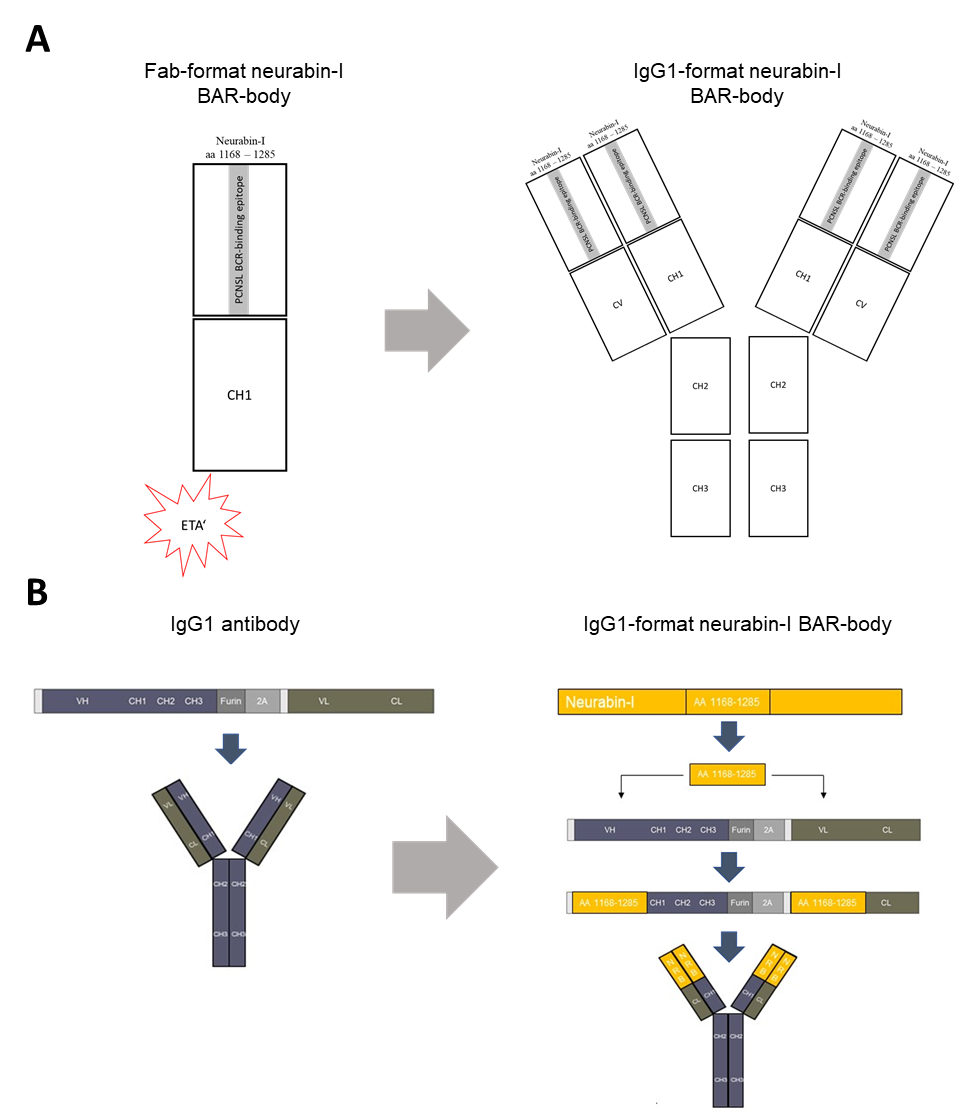


***Supplemental figure 6:*** (A) Sketches of both BAR-body products. The left side shows the heavy-chain-only Fab-format neurabin-I BAR body in version B (see Suppl. fig. 1). The right side depicts the IgG1-format BAR-body integrating neurabin-I epitopes in exchange for variable regions. (B) Cloning strategy for the IgG1-format neurabin-I BAR-body. A pSfi FLAG-Tag expression vector containing an IgG1 sequence (VH, CH1-CH3, Furin + 2A sequence, VL, CL) was used as template. VH and VL were exchanged with a 120 amino acids sequence of neurabin-I (aa 1168 – 1285) containing the PCNSL reactive epitope (aa 1226 – 1251). The amino acid sequence enumeration refers to isoform 3 of neurabin-I (https://www.uniprot.org).
